# Untangling cross-regional cross-frequency coupling in dynamic neural oscillations

**DOI:** 10.1088/1741-2552/ae2293

**Published:** 2025-12-02

**Authors:** Soroush Niketeghad, Koorosh Mirpour, Mahsa Malekmohammadi, Evangelia Tsolaki, Nader Pouratian, William Speier

**Affiliations:** 1 Department of Bioengineering, University of California, Los Angeles, CA, United States of America; 2 Department of Neurological Surgery, UT Southwestern Medical Center, Dallas, TX, United States of America; 3 Department of Neurosurgery, University of California, Los Angeles, CA, United States of America; 4 Department of Radiological Sciences, University of California, Los Angeles, CA, United States of America

**Keywords:** cross-frequency coupling, phase coupling, causality, thalamocortical, neural oscillation

## Abstract

*Objective.* Brain networks communicate through long range phase coupling of low frequency oscillations (LFOs, less than 35 Hz) between brain regions. At the same time, phase-amplitude cross-frequency coupling (CFC), in which the phase of the same LFO have been shown to modulate the power of high frequency activity has also been reported across brain regions as a critical regulator of neural activity and excitability. While cross-regional CFC has been reported as a potential mechanism of long-distance modulation of neural excitability, the mechanism underlying this phenomenon has yet to be understood and methods to dissociate the effect of local vs remote LFO have not been developed. Cross-regional CFC can be a result of either LFOs in one region directly modulating high frequency oscillations in another region or due to a chain effect, in which apparent cross-regional CFC results from coupling of LFO across sites. *Approach.* A novel method of partial modulation index (PMI) is proposed as a derivation of modulation index (MI) and based on Pearl’s do-calculus to remove the mathematical bias of simultaneous phase coupling and CFC measurements. Here, we first test the PMI on a simulated dataset, showing it can differentiate between biased and unbiased CFC. We then evaluate the method on intracranially collected local field potentials recorded simultaneously from thalamus and cortex in a patient undergoing deep brain stimulator implantation for essential tremor, demonstrating that the observed thalamocortical CFC was partially biased. *Main results.* For both simulated and human datasets, the PMI was compared to the conventional MI. In simulated data, the PMI was able to disentangle cross-regional phase coupling and focal CFC which is not possible using conventional MI. While there is no ground truth for comparison in human data, the results from the simulated data demonstrate the value of the proposed method in removing mathematical bias. *Significance.* This novel method facilitates a mathematically rigorous characterization of residual CFC, enabling investigations of differential contributions and roles of brain-wide LFO to CFC, which can lead to a more complete understanding of the pathophysiology of neurological processes and disorders.

**List of Abbreviations**
CFC: cross-frequency coupling
LFO: low frequency oscillation
HFA: high frequency activity
HFO: high frequency oscillation
SFC: same-frequency coupling
ET: essential tremor
DAG: directed acyclic graph
PCFC: partial cross-frequency coupling
MI: modulation index
PMI: partial modulation index
DBS: deep brain stimulation
ECoG: electrocorticogram
ViM: ventral intermediate nucleus
M1: primary motor cortex

## 1. Introduction

Oscillations reflect the rhythmic modulation of excitability in neural populations where each oscillatory cycle provides a short window of maximum excitability of activity in a distinct frequency band (Lakatos *et al* 2005, Jensen and Colgin 2007, Canolty and Knight 2010). The ‘nesting’ of oscillations can be measured via CFC between a *modulating* LFO and *modulated* HFA of a signal (Canolty *et al* 2010). CFC has been demonstrated in various task-related processes including attention (Sauseng *et al* 2008), learning (Tort *et al* 2009), and emotional regulation (Popov *et al* 2012) as a dynamic regulatory mechanism of cortical activity (Malekmohammadi *et al* 2015). In addition to the focal nesting behavior, oscillations can synchronize across distant brain regions enabling selective and dynamic control of distributed functional cell assemblies (Varela *et al* 2001, Fries 2005). This functional connectivity between task-relevant regions is established by SFC of LFO phases (Jensen and Mazaheri 2010, Siegel *et al* 2012), and can be complemented with measures of *effective* connectivity (Friston *et al* 2013) such as Granger causality (Bernasconi and König 1999, Liang *et al* 2000, Hesse *et al* 2003, Brovelli *et al* 2004, Chen *et al* 2006) to determine the direction of information transmission.

If a particular oscillation is simultaneously involved in both local CFC and cross-regional SFC, a cross-regional CFC can be measured as a byproduct. For instance, cross-regional CFC between the phase of thalamic LFO and amplitude of cortical HFA has been reported in ET patients using intraoperative recordings (Opri *et al* 2019). Focal cortical CFC and thalamocortical SFC were present and were shown to peak in the same frequency ranges for each patient. These couplings were diminished during movement suggesting a potential responsive mechanism for motor suppression triggered by the thalamus. However, to be able to draw causal conclusions, a temporally sensitive analysis is required to account for the potential interdependencies of SFC and CFC. Unlike cross-regional SFC and focal CFC, we do not have a distinct physiological explanation for cross-regional CFC. A network-level connectivity analysis is needed to control for the potential bias of latent nodes in order to disentangle these signals, allowing for more targeted analysis of coupling pathways. To drive a causal conclusion of one node triggering a mechanism, an effective connectivity analysis is essential to assess the direction of information flow.

In the case of observed thalamocortical CFC, we hypothesize if LFO flows from cortex to the thalamus, cortical LFO would have a confounding bias on the measured thalamocortical CFC. In other words, observed thalamocortical CFC can at least partially be a byproduct of cortical LFO modulating thalamic LFO and cortical HFA.

Here, we develop a causal model of cross-regional CFC illustrated as a DAG. Inspired by Judea Pearl’s work in economics (Pearl 2003), we introduce a novel method to evaluate PCFC to account for the potential confounding bias of SFC. To test our method against dynamic variation connectivity measures, simulated time series are used to distinguish between simultaneous and non-overlapping occurrences of SFC and cross-regional and focal CFC. Finally, we use PCFC on simultaneously acquired intra-operative LFP recordings from both motor cortex and thalamus in an ET patient along with an estimation for Granger causality to demonstrate an application of PCFC in untangling the dynamics of thalamocortical CFC.

## 2. Methods

### 2.1. Background

#### 2.1.1. DAGs and Pearl’s do-calculus

**Figure 1.**
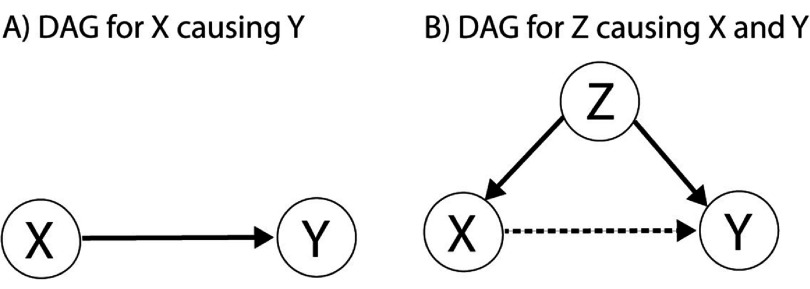
Directed acyclic graph used to illustrate the potential model based on the observed causal relationship from *X* to *Y*. (A) A simple assumption of *X* causing *Y*. (B) A latent parent node *Z* is causing *X* and *Y* which led to observed association between *X* and *Y* (dotted line).

DAG displays causal assumptions (directed edges in the context of graph theory) about the causal relationships among variables (nodes) through sequences of direct edges (paths with the length of the number of edges) where there is no path from a node to itself (cycle) (Suttorp *et al* 2015). Let us assume we measure two variables *X* and *Y* and observe a causal relationship from *X* on *Y*. This relationship can be illustrated as a DAG with two nodes and a direct edge from node *X* to node *Y* (figure 1(A)). We can also use the language of probability theory to describe this causal relationship as ${\text{Pr}}\left( {y|x} \right) \ne {\text{Pr}}\left( y \right)$ where ${\text{Pr}}\left( y \right)$ is the probability of outcome *Y* = *y* and ${\text{Pr}}\left( {y|x} \right)$ is the conditional probability of outcome *Y* = *y* given the occurrence of outcome *X* = *x*.

A *latent* node *Z* with directed edges going to both *X* and *Y* (figure 1(B)) that was not incorporated in our initial observation, forms a fork (*X* ← *Z* → *Y*) which introduces a confounding bias on *X* → *Y*. In other words, the observed causal effect from *X* on *Y* could be entirely (or partially) due to effects of parent node *Z* on its children *X* and *Y*. In fact, a direct causal effect of *X* on *Y* may not exist or may be weaker than expected. Judea Pearl, who developed much of the theory of causal graphs, addresses the absence in probability language of a way to distinguish setting a variable from observing it by inventing *do-calculus* (Pearl 2003). He introduces ${\text{do}}\left( x \right)$ for setting *X = x*, and *x* for observing *X = x*. Thus expression ${\text{Pr}}\left( {y|{\text{do}}\left( x \right)} \right)$ means to delete all the arrows going to *x* when finding the conditional probability of *y* given *x*. Thus if ${\text{Pr}}\left( {y|x} \right) \ne {\text{Pr}}\left( {y|{\text{do}}\left( x \right)} \right)$, there is a confounding bias. He also defines *back-door criterion* for node *Z* relative to *X* → *Y* as it to not be a descendant of neither *X* nor *Y* and it blocks every path between *X* and *Y* that contains an arrow into *X*. Pearl then proves that if *Z* satisfies the back-door criterion relative to *X* → *Y* then ${\text{Pr}}\left( {y|{\text{do}}\left( x \right)} \right) = \mathop \sum \limits_z^{ } {\text{Pr}}\left( {y|x,z} \right){\text{Pr}}\left( z \right)$ which can be used to estimate ${\text{Pr}}\left( {y|{\text{do}}\left( x \right)} \right)$ based on estimations of ${\text{Pr}}\left( {y|x,z} \right)$ and ${\text{Pr}}\left( z \right)$ observations.

##### 2.1.2. Modulation index (MI)

MI proposed by Tort and colleagues (Tort *et al* 2010) is one of the common measures of CFC which quantifies phase amplitude coupling (PAC). MI discretizes the phase angle time series of the modulating frequency $\phi \left( t \right)$ into *N* phase bins, calculates the mean of the amplitude time series of the modulated frequency $A\left( t \right)$ in each bin, and normalizes mean amplitude $\langle A\rangle \left[ i \right]$ by dividing each bin value by the sum over the bins:

\begin{equation*}P\left[ i \right] = \frac{{\langle A\rangle \left[ i \right]}}{{{{\mathop \sum \nolimits}}_{k = 1}^N\langle A\rangle \left[ k \right]}}.\end{equation*}

The normalized amplitude *P* has the characteristics of a discrete probability density function and if there is no coupling between $\phi $ and *A, P* would be a uniform distribution. Thus Kullback–Leibler (KL) distance is used to measure the statistical deviation of *P* from uniform distribution *U*:

\begin{equation*}{D_{{\text{KL}}}}\left( {P,U} \right) = \mathop \sum \limits_{i = 1}^N P\left[ i \right]{\text{log}}\left( {\frac{{P\left[ i \right]}}{{U\left[ i \right]}}} \right){ }{\text{}}.\end{equation*}

KL distance can also be written as

\begin{equation*}{D_{{\text{KL}}}}\left( {P,U} \right) = \log \left( N \right) - H\left( P \right),\end{equation*}

where *H* represents the Shannon entropy. Given that ${\text{log}}\left( N \right)$ is the maximum possible entropy value, MI is defined by normalizing the KL distance between *P* and *U*:

\begin{align*}{\text{MI}}\left( {\phi ,A} \right) = \frac{{{D_{{\text{KL}}}}\left( {P,U} \right)}}{{{\text{log}}\left( N \right)}}.\end{align*}

#### 2.1.3. Proposed method: PMI

While MI was originally proposed to measure local PAC between two frequency components of a signal, it can also measure the cross-regional PAC. Hence, we adapt MI to incorporate the confounding bias of the phase of modulating frequency in focal CFC and introduce PMI as a measure of cross-regional CFC while controlling for the effects of the focal phase.

**Figure 2.**
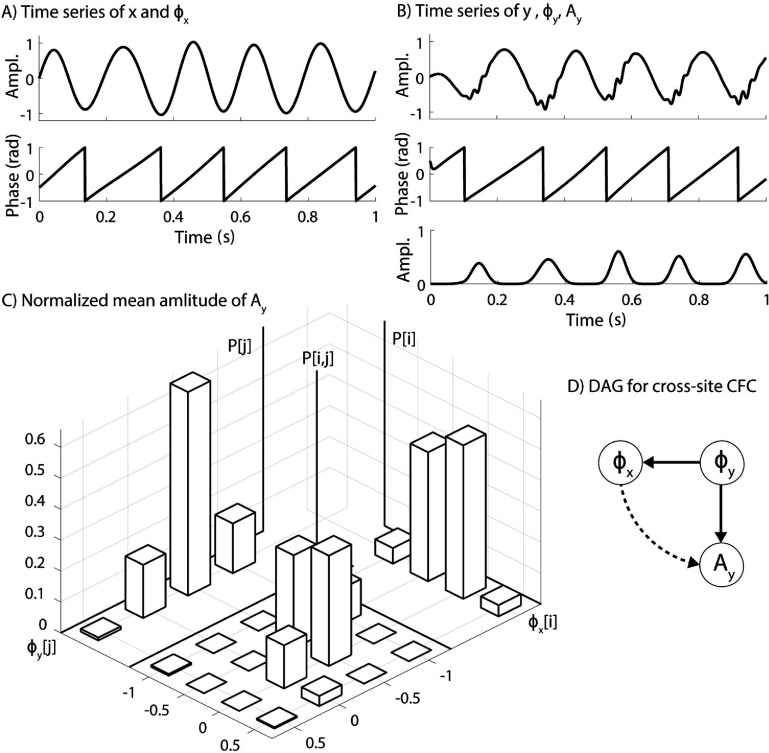
Steps towards calculation of partial modulation index between the phase *φ_x_* of the modulating frequency of signal *x* and amplitude *A_y_* of the modulated frequency of signal *y*. (A) Time series of *x* and the phase of its modulating frequency (*φ_x_*). (B) Time series of signal *y*, the phase of its modulating frequency (*φ_y_*), and the amplitude of its modulated frequency (*A_y_*). Normalized mean amplitude of *A_y_* over the phase bins of *φ_x_*[*i*] (*P*[*i*]), phase bins of *φ_y_*[*j*] (*P*[*j*]) and the two-dimensional phase bins of (*φ_x_*[*i*], *φ_y_*[*j*]) (*P*[*i,j*]). (D) The directed acyclic graph for the casual coupling from *φ_y_* to both *φ_x_* and *A_y_* (solid arrows) and the observed coupling from *φ_x_* to *A_y_* (dotted arrow).

*Assumption.* We assume that CFC always represents a causal flow from the modulating phase to the modulated amplitude. The cross-regional CFC under study is observed between the phase of a modulating frequency ${\phi _x}$ of signal *x* (figure 2(A)) and the amplitude of the modulated frequency ${A_y}$ of signal *y* (figure 2(B)). Here we also introduce a parent node ${\phi _y}$ which represents the phase of the modulating frequency of signal *y*. In addition to the observed ${\phi _x} \to {A_y}$ coupling, we have determined the existence of a focal CFC in *y* (${\phi _y} \to {A_y}$) as well as a causal cross-regional SFC (${\phi _y} \to {\phi _x}$) which are physiologically established as direct causal relationships (figure 2(D)).

*Goal.* We aim to remove the confounding bias of ${\phi _y}$ by removing the (${\phi _y} \to {\phi _x}$) while calculating the MI for ${\phi _x} \to {A_y}$.

*Method.* When calculating MI between *x* and *y*, the normalized amplitude $\langle {A_y}\rangle \left[ i \right]$ represents an estimation of the expected value of ${A_y}$ for each phase bin *i* of ${\phi _x}$ ($E\left( {{A_y}|{\phi _x}\left[ i \right]} \right)$ and can be written as

\begin{align*}E\left( {{A_y}|{\phi _x}\left[ i \right]} \right) = \mathop \sum \limits_{{A_y}}^{ } {A_y}{\text{Pr}}\left( {{A_y}|{\phi _x}\left[ i \right]} \right),\end{align*}

where ${\text{Pr}}\left( {{A_y}|{\phi _x}\left[ i \right]} \right)$ represents the conditional probability of amplitude values of ${A_y}$ occurring during phase bin values of ${\phi _x}\left[ i \right]$.

Using Pearl’s do-calculus, we can isolate the effect of ${\phi _y}$ on ${A_y}$ by using the do operator on ${\phi _x}$ and since ${\phi _y}$ satisfies the back-door criterion relative to ${\phi _x} \to {A_y}$

\begin{equation*}\begin{gathered} E\left( {{A_y}|{\phi _x}\left[ i \right]} \right) = \sum\limits_{{A_y}} {{A_y}} {\text{Pr}}\left( {{A_y}|{\phi _x}\left[ i \right]} \right) \hfill \\ \sum\limits_{{A_y}} {{A_y}} \mathop \sum \limits_{j = 1}^N {\text{Pr}}\left( {{\phi _y}\left[ j \right]|{\phi _x}\left[ i \right]} \right)P\left( {{A_y}|{\phi _y}\left[ j \right],{\phi _x}\left[ i \right]} \right) \hfill \\ \sum\limits_{{A_y}} {{A_y}} \mathop \sum \limits_{j = 1}^N {\text{Pr}}\left( {{\phi _y}\left[ j \right]} \right){\text{Pr}}\left( {{A_y}|{\phi _y}\left[ j \right],{\phi _x}\left[ i \right]} \right) \hfill \\ \mathop \sum \limits_{j = 1}^N {\text{Pr}}\left( {{\phi _y}\left[ j \right]} \right)\sum\limits_{{A_y}} {{A_y}{\text{Pr}}\left( {{A_y}|{\phi _y}\left[ j \right],{\phi _x}\left[ i \right]} \right)} \hfill \\ \mathop \sum \limits_{j = 1}^N {\text{Pr}}\left( {{\phi _y}\left[ j \right]} \right)E\left( {{A_y}|{\phi _y}\left[ j \right],{\phi _x}\left[ i \right]} \right) \hfill \\ \end{gathered} \end{equation*}

where ${\text{Pr}}\left( {{\phi _y}\left[ j \right]} \right)$ can be estimated as the normalized histogram of ${\phi _y}$ over phase bins and $E\left( {{A_y}|{\phi _y}\left[ j \right],{\phi _x}\left[ i \right]} \right)$ can be estimated as the mean amplitude for the common outcome of $\left( {{\phi _y}\left[ j \right],{\phi _x}\left[ i \right]} \right)$ or $\langle A\rangle \left[ {i,j} \right]$ as illustrated in figure 2(C). Therefore, the normalized mean amplitude $P^{\prime}$ can be calculated as

\begin{align*}P{^{^{\prime}}}\left[ i \right] = \frac{{{{\mathop \sum \nolimits}}_{j = 1}^N{\text{Pr}}\left( {{\phi _y}\left[ j \right]} \right)\langle {A_y}\rangle \left[ {i,j} \right]}}{{{{\mathop \sum \nolimits}}_{k = 1}^N\langle {A_y}\rangle \left[ k \right]}},\end{align*}

and PMI is defined as

\begin{align*}{\text{PMI}}\left( {{\phi _x},{A_y}} \right) = \frac{{{D_{{\text{KL}}}}\left( {P{^{^{\prime}}},U} \right)}}{{{\text{log}}\left( N \right)}}.\end{align*}

### 2.2. Evaluation methods

#### 2.2.1. Verification using simulated data

##### 2.2.1.1. Dataset

**Figure 3.**
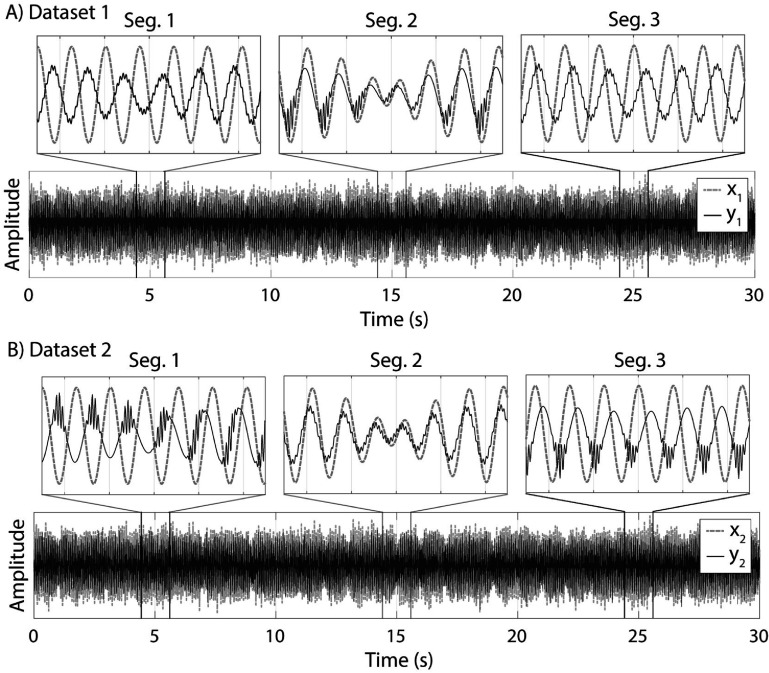
Simulated time series generated for testing the proposed method. (A) Dataset 1 containing x_1_ and y_1_ with phase coupling at 13 Hz and cross-regional and focal CFC between 13 Hz and 150 Hz only for Seg. 2. (B) Dataset 2 containing x_2_ and y_2_ with cross-regional CFC between 13 Hz and 150 Hz for Seg. 1, phase coupling at 13 Hz for Seg. 2, and focal CFC between 13 Hz and 150 Hz for Seg. 3. Note that for illustration purposes, the zoomed in figures for each segment do not include the added noise n(t).

To evaluate the sensitivity of our method to dynamic CFC and SFC relations, we generated two sets of simulated time series data $\left\{ {{x_i}\left( t \right),{y_i}\left( t \right)} \right\}$ for $j = \left\{ {1,2} \right\}$ with length of $T = 30\,{\text{s}}$ and sampling rate of ${f_{\text{s}}} = 1\,{\text{kHz}}$. ${x_i}$ contained a low frequency (${f_{\text{l}}}$) sine wave as the modulating frequency component, and ${y_i}$ contained a low frequency (${f_{\text{l}}}$) and a high frequency (${f_{\text{h}}} = 150\,{\text{Hz}}$) sine wave as modulating and modulated frequency components. ${f_{\text{l}}}$ was a random variable with normal distribution ($\mu = 13\,{\text{Hz}},\sigma = 1\,{\text{Hz}}$) and was used to couple (same seed) or decouple (different seeds) the frequency components. As shown in figure 3(A), $\left\{ {{x_1}\left( t \right),{y_1}\left( t \right)} \right\}$ were simultaneously coupled between the phase of ${x_1}$ and phase of ${y_1}$ at ${f_{\text{l}}}$, and phase of ${y_1}$ at ${f_{\text{l}}}$ with amplitude of ${y_1}$ at ${f_{\text{h}}}$ only for a time segment of $\frac{T}{3} \unicode{x2A7D} t &lt; \frac{{2T}}{3}$:

\begin{align*} \left\{ {\begin{array}{*{20}{l}} {x_1} = \sin\left( {2\pi {f_{\text{l}}}\left( {t + {\tau _1}} \right)} \right) + n\left( t \right), \\ {y_1} = \sin\left( {2\pi {f_{\text{l}}}\left( {t + {\tau _2}} \right)} \right) + k\sin\left( {2\pi {f_{\text{h}}}t} \right) + n\left( t \right)\;\;\;\;\;\;\;\;\;\;\;\;\;\;\;\;\;\;\;\;\;\;\;\;\;\;\;\;\;\;\;\;\;\;\;\;\;\;\;\;\;\;\;\;\;\;\;\;\;\;\;\;\;\;\;\;\;0 \unicode{x2A7D} t &lt; \frac{T}{3} \\ {x_1} = \sin\left( {2\pi {f_{\text{l}}}\left( {t + {\tau _1}} \right)} \right) + n\left( t \right), \\ {y_1} = \sin\left( {2\pi {f_{\text{l}}}\left( {t + {\tau _1}} \right) + \varphi } \right) + k\sin\left( {2\pi {f_{\text{l}}}\left( {t + {\tau _1}} \right) + \varphi } \right)\sin\left( {2\pi {f_{\text{h}}}t} \right) + n\left( t \right)\;\;\;\;\;\;\;\;\;\frac{T}{3} \unicode{x2A7D} t &lt; \frac{{2T}}{3} \\ {x_1} = \sin\left( {2\pi {f_{\text{l}}}\left( {t + {\tau _2}} \right)} \right) + n\left( t \right), \\ {y_1} = \sin\left( {2\pi {f_{\text{l}}}\left( {t + {\tau _1}} \right)} \right) + k\sin\left( {2\pi {f_{\text{h}}}t} \right) + n\left( t \right)\;\;\;\;\;\;\;\;\;\;\;\;\;\;\;\;\;\;\;\;\;\;\;\;\;\;\;\;\;\;\;\;\;\;\;\;\;\;\;\;\;\;\;\;\;\;\;\;\;\;\;\;\;\;\;\;\frac{{t \unicode{x2A7E} 2T}}{3} \end{array}} \right..\end{align*}

where ${\tau _1}$, ${\tau _2}$ are random variables with normal distribution ($\mu = 0,\,\sigma = \frac{1}{{{f_{\text{l}}}}}$) that allowed us to implement random seeding for ${f_{\text{l}}}$ and $n\left( t \right)$ is a time series of random noise with normal distribution ($\mu = 0,\sigma = 1$). In this case, we would expect to observe a cross-regional CFC while cross-regional PCFC would be insignificant as the periods of cross-regional phase coupling and focal CFC completely overlap.

On the other hand, as shown in figure 3(B) for $\left\{ {{x_2}\left( t \right),{y_2}\left( t \right)} \right\}$, we coupled the phase of ${x_2}$ at ${f_{\text{l}}}$ with amplitude of ${y_2}$ at ${f_{\text{h}}}$ for $0 \unicode{x2A7D} t &lt; \frac{T}{3}$, phase of ${x_2}$ and phase of ${y_2}$ at ${f_{\text{l}}}$ for $\frac{T}{3} \unicode{x2A7D} t &lt; \frac{{2T}}{3}$, and phase of ${y_2}$ at ${f_{\text{l}}}$ with amplitude of ${y_2}$ at ${f_{\text{h}}}$ for $\frac{{t \ge 2T}}{3}$ so that cross-regional CFC, cross-regional phase coupling and focal CFC did not overlap

\begin{align*}\left\{ {\begin{array}{*{20}{l}} {x_2} = \sin\left( {2\pi {f_l}\left( {t + {\tau _1}} \right)} \right) + n\left( t \right), \\ {y_2} = \sin\left( {2\pi {f_l}\left( {t + {\tau _2}} \right) + \varphi } \right) + k\sin\left( {2\pi {f_l}\left( {t + {\tau _1}} \right) + \varphi } \right)\sin\left( {2\pi {f_{\text{h}}}t} \right) + n\left( t \right)\;\;\;\;\;\;\;\;\;\;\;\;\;\;\;\;\;0 \le t &lt; \frac{T}{3} \\ {x_2} = \sin\left( {2\pi {f_{\text{l}}}\left( {t + {\tau _1}} \right)} \right) + n\left( t \right), \\ {y_2} = \sin\left( {2\pi {f_{\text{l}}}\left( {t + {\tau _1}} \right)} \right) + k\sin\left( {2\pi {f_{\text{h}}}t} \right) + n\left( t \right)\;\;\;\;\;\;\;\;\;\;\;\;\;\;\;\;\;\;\;\;\;\;\;\;\;\;\;\;\;\;\;\;\;\;\;\;\;\;\;\;\;\;\;\;\;\;\;\;\;\;\;\;\;\;\;\;\;\;\;\;\;\;\;\;\;\frac{T}{3} \le t &lt; \frac{{2T}}{3} \\ {x_2} = \sin\left( {2\pi {f_{\text{l}}}\left( {t + {\tau _1}} \right)} \right) + n\left( t \right), \\ {y_2} = \sin\left( {2\pi {f_{\text{l}}}\left( {t + {\tau _2}} \right) + \varphi } \right) + k\sin\left( {2\pi {f_{\text{l}}}\left( {t + {\tau _2}} \right) + \varphi } \right)\sin\left( {2\pi {f_{\text{h}}}t} \right) + n\left( t \right)\;\;\;\;\;\;\;\;\;\;\;\;\;\;\;\;\frac{{t \ge 2T}}{3} . \end{array}} \right..\end{align*}

In this case, we would expect to observe a significant cross-regional CFC as well as cross-regional PCFC due to non-overlapping periods of cross-regional CFC, cross-regional phase coupling, and focal CFC.

To further test the method’s robustness under more realistic conditions, we also generated two more datasets as explained above but with LFP-like signals. These signals were constructed with three key features to mimic the characteristics of biological data: a primary sinusoidal oscillation at the target frequency (13 Hz), a scale-free aperiodic noise component, and broadband noise. The aperiodic component, which mimics the characteristic 1/*f* power spectrum of biological signals, was created by summing 1000 sinusoids with random frequencies (1–200 Hz) and amplitudes inversely proportional to their frequency. Additionally, Gaussian white noise was added to represent stochastic neural activity. The final LFP-like signal was normalized to an RMS of 1 and combined with a modulated high-frequency component to construct the final time series. The rest of the methodology for LFP-like signal datasets 1 and 2 was the same as above for sinusoidal signals.

##### 2.2.1.2. Analysis

CFC and PCFC were estimated by calculating MI and PMI respectively with identical parameters. A zero-phase FIR bandpass filter (Delorme and Makeig 2004) followed by Hilbert transform was used to obtain instantaneous phase (between 2 Hz and 35 Hz) and amplitude (between 3 Hz and 250 Hz) of the signals. Band width for calculating the instantaneous phase was set to 2 Hz while for instantaneous amplitude bandwidth was adjusted as twice the phase of interest to fit the sidebands caused by the modulating frequency band (Aru *et al* 2015). Phases were divided into *N* = 18 bins (bin width = 2$\pi $/*N*) for both MI (a vector of length 18) and PMI (a matrix of 18 by 18 elements). A surrogate data (*N* = 1000) was produced by randomly shifting the amplitude time series of the modulated frequency in the time domain and non-parametric permutation test was used to assess the statistical significance of MI and PMI by calculating a *p* value for each pair of phase and amplitude frequency. A cluster-based statistics was used to correct for the multiple comparison in the two-dimensional domain of the modulating and modulated frequencies (Maris and Oostenveld 2007).

#### 2.2.2. Application to human data

##### 2.2.2.1. Dataset

Data was recorded from a 63-year-old female undergoing stereotactic implantation of DBS lead (Medtronic 3389) for treatment of tremor. Patient provided written informed consent approved by the Institutional Review Board at the University of California Los Angeles and the research was conducted in accordance with the principles embodied in the Declaration of Helsinki and in accordance with local statutory requirements. Before implantation of the DBS lead, an eight-contact subdural ECoG strip (platinum–iridium 4 mm contacts with 1 cm spacing; AdTech Medical, USA) was implanted posteriorly towards the central sulcus through the frontal burr hole placed for DBS lead implantation (Malekmohammadi *et al* 2019). DBS electrode implantation targeted right ViM and intraoperative awake macrostimulation testing was performed to confirm the clinically valid placement of implanted DBS leads with tremor suppression. Location of DBS electrodes and the ECoG strip was assessed using a single-view lateral fluoroscopy image captured after implantation of the DBS lead and postoperative stereotactic thin slice CT (DBS lead only). The ECoG strip was removed after conducting the experiments, prior to anchoring the DBS lead.

Data was recorded using g.USBamp 2.0 amplifiers (g.Tec, Austria) and BCI2000 data acquisition software with ground and reference electrodes connected to the scalp. The sampling frequency was 2400 Hz and the data was band pass filtered (0.1–1000 Hz) in real time. Patient performed 3 blocks of finger tapping hand movement initiated by a verbal cue, using the hand contralateral to the ECoG strip placement. Each movement block lasted ∼30 s and was preceded by a 30 s period of rest with eyes open. Here, we separately analyze the resting state and movement data by concatenating the three available blocks for each phase of the experiment. Post-surgery, we extracted the ViM recordings by bipolar re-referencing the data recorded from the two most ventral contacts (0–1) of the DBS lead. ECoG contacts were localized relative to a 3D-reconstructed cortical surface by coregistering pre-op MRI, pre-op and post-op CT scans, and an x-ray scan performed prior to extraction of the ECoG strip. The M1 recording was extracted by bipolar re-referencing the data recorded from the two neighboring contacts across the central sulcus. An adaptive notch filter was used to remove the power line noise and its harmonics.

##### 2.2.2.2. Analysis

The calculations of MI, PMI, and corresponding statistical analysis were identical to what was described in the previous section for the simulated dataset. To determine the causality of SFCs we incorporated Granger causality into our analysis. Granger causality was estimated by calculating DTF of the system of the two time series based on a MVAR model of the signals in the frequency range of 2–35 Hz using Matlab EConnectome toolbox (He *et al* 2011). A non-parametric permutation test was used to assess the *p* values for each frequency based on surrogate data (*N* = 1000) produced by randomizing data points in the frequency domain. Under the assumption that there are at most 5 independent frequency bands in the range of 2–35 Hz (delta, theta, alpha, low and high beta) and given that DTF was calculated both ways, the significance threshold was set to 0.05/10 = 0.005 based on Bonferroni correction for multiple comparison.

To validate the Granger causality findings with a method based on different principles, we also calculated transfer entropy (TE). We modified the algorithm introduced by (Lobier *et al* 2014) to quantify the direction of information flow between VIM and M1. Continuous phase and amplitude signals were discretized into 18 uniformly spaced bins. TE was then calculated based on the joint and conditional probabilities of these binned states, using a lag of 1 sample. The final TE value, expressed in bits, quantifies the information transferred from the source to the target. TE was calculated for each low-frequency phase band in both directions: VIM to M1 and M1 to VIM.

## 3. Results

### 3.1. Simulated data

**Figure 4.**
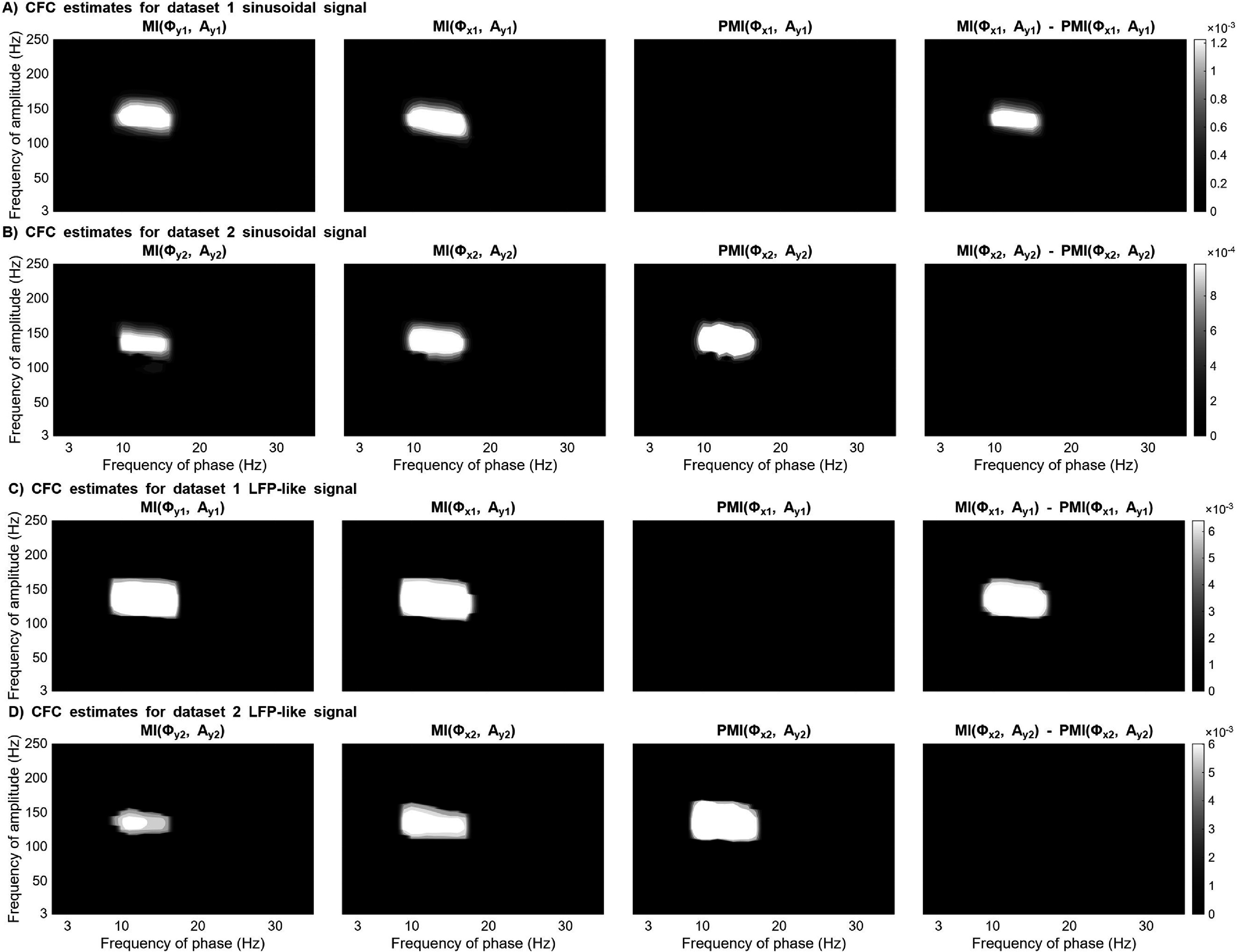
Performance of two cross-frequency coupling (CFC) estimation methods on simulated datasets. This figure compares comodulograms generated using the standard modulation index (MI) and a proposed method (PMI). The methods were tested on a simple sinusoidal signal (A), (B) and a more complex LFP-like signal (C), (D). To highlight true effects, non-significant coupling values (p ⩾ 0.05) were removed using cluster-corrected permutation testing.

In figure 4, focal (${\text{MI}}\left( {{\phi _y},{A_y}} \right)$) and cross-regional (${\text{MI}}\left( {{\phi _x},{A_y}} \right)$) CFC can be recognized between the modulating frequency (13 Hz) and modulated frequency (150 Hz) for both datasets. As expected for dataset 1, ${\text{PMI}}\left( {{\phi _{x1}},{A_{y1}}} \right)$ did not pass the significance threshold and the difference between ${\text{MI}}\left( {{\phi _{x1}},{A_{y1}}} \right)$ and ${\text{PMI}}\left( {{\phi _{x1}},{A_{y1}}} \right)$ is recognizable (figure 4(A)). On the other hand for dataset 2, ${\text{PMI}}\left( {{\phi _{x2}},{A_{y2}}} \right)$ can be recognized and there is no significant difference between ${\text{MI}}\left( {{\phi _{x2}},{A_{y2}}} \right)$ and ${\text{PMI}}\left( {{\phi _{x2}},{A_{y2}}} \right)$ (figure 4(B)).

**Figure 5.**
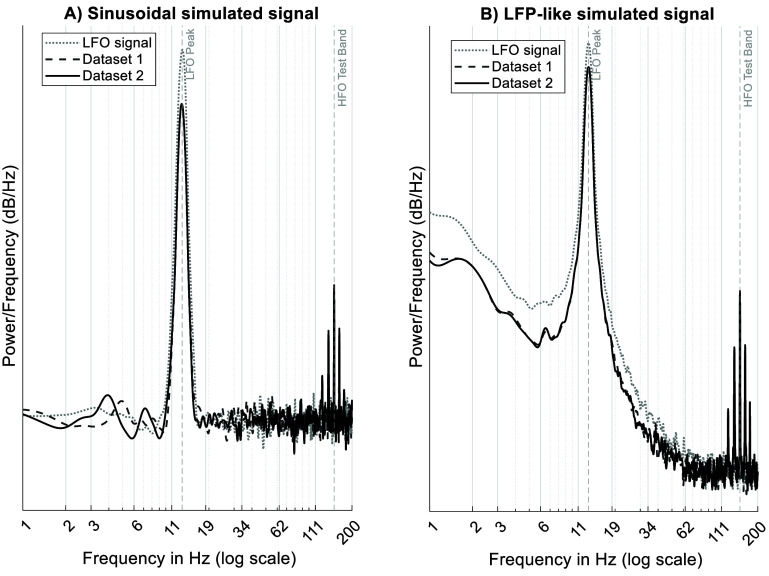
Power spectra of the simulated signals used to evaluate the CFC estimation methods. (A) The power spectral density for signals created with a pure sinusoidal waveform. (B) The power spectral density for signals designed to mimic the non-sinusoidal, noisy.

To validate our method on more physiologically plausible signals, we repeated the simulation using LFP-like data containing aperiodic and broadband noise. These results replicated our primary findings (figures 4(C) and (D)), confirming that PMI effectively differentiates between confounded and true CFC even when signals are not strictly narrowband. Figure 5 compares the spectrogram of the sinusoidal simulated signal (panel (A)) and LFP-like signal (panel (B)).

### 3.2. Sensitivity analysis

**Figure 6.**
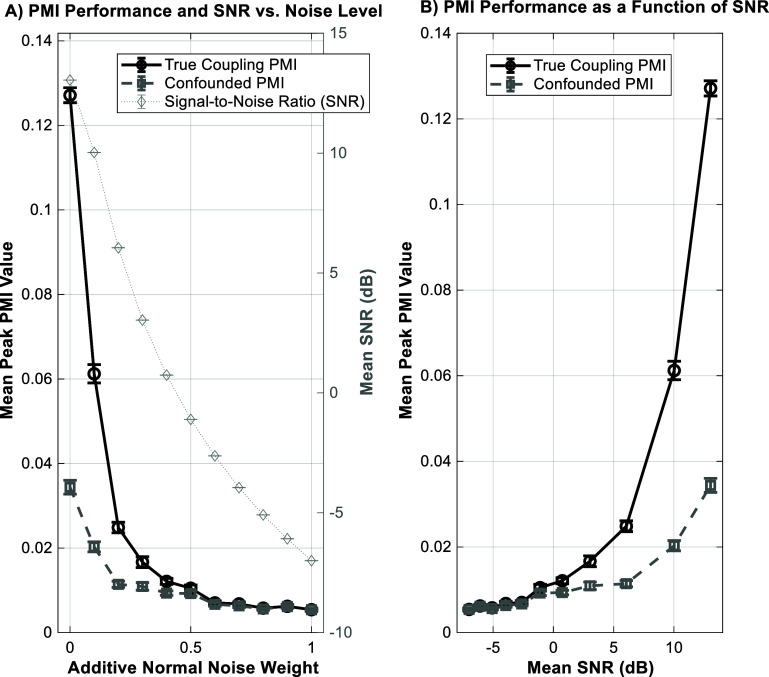
Robustness of the PMI algorithm to noise. Results from a Monte Carlo simulation show the performance of the PMI algorithm. (A) As the level of additive noise increases, the measured PMI for true coupling (black solid line) decreases, along with the corresponding signal-to-noise ratio (SNR, gray dotted line). The PMI for confounded coupling (gray dashed line) remains near zero. (B) Plotting PMI as a function of SNR demonstrates that the algorithm reliably detects true coupling at higher SNRs while consistently rejecting confounded coupling across all noise levels.

**Figure 7.**
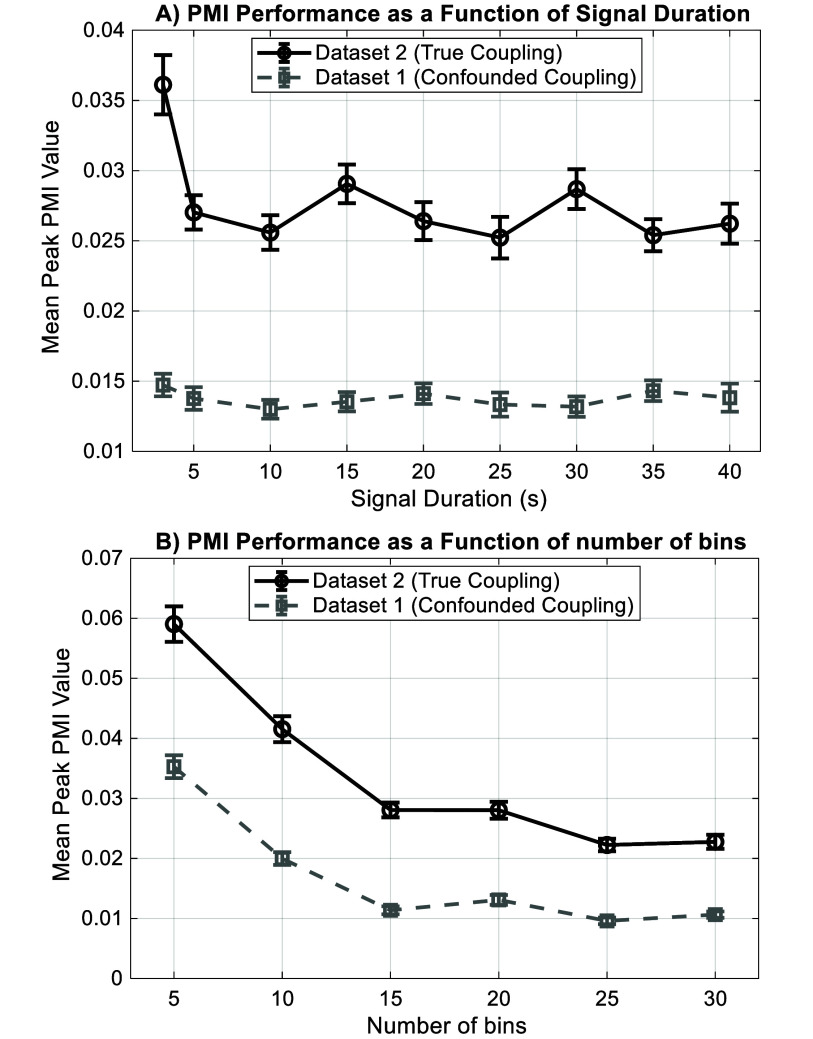
Sensitivity of the PMI algorithm to signal duration and bin count. (A) PMI performance as a function of signal duration. The algorithm maintains a stable and robust distinction between true coupling (black solid line) and confounded coupling (gray dashed line) for signal lengths of 10 s and longer. (B) PMI performance as a function of the number of phase bins.

To assess the robustness and practical limitations of our method, we performed a series of Monte Carlo simulations (see methods). The PMI algorithm successfully differentiated true (dataset 2) from confounded (dataset 1) CFC even at low signal-to-noise ratios (figure 6). Furthermore, the method demonstrated stable performance across a wide range of signal durations, confirming that the 30 s epoch used in our main analysis is sufficient for reliable estimation (figure 7(A)). Finally, our choice of 18 phase bins was shown to fall within a stable range of 15–30 bins, indicating that the results are not critically sensitive to this parameter (figure 7(B)).

### 3.3. Human data

**Figure 8.**
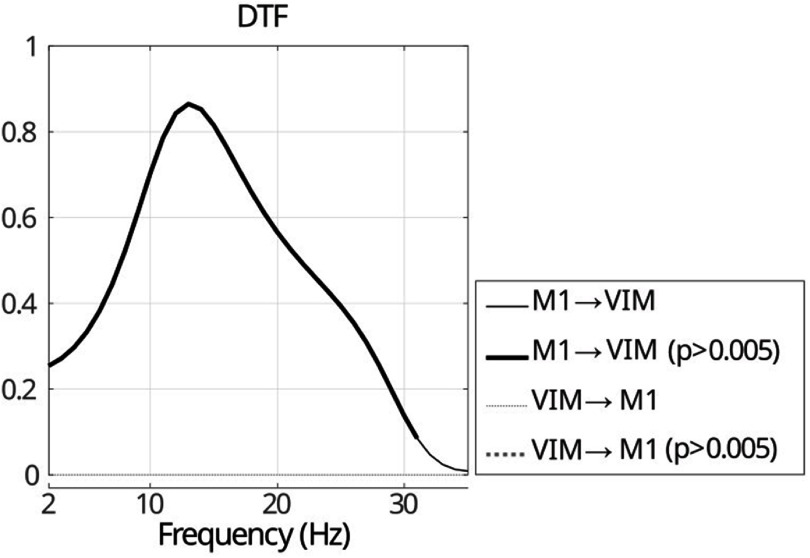
Estimation of granger causality for both directions of VIM-M1 coupling using direct transfer function. Significant DTF values for each frequency based nonparametric permutation testing and Bonferroni correction for multiple comparison (p < 0.005) are highlighted with a heavy line (VIM to M1 coupling is always zero and insignificant).

A significant flow from M1 to VIM in the frequency range of 2–31 Hz while the VIM to M1 flow is insignificant for the whole frequency range of interest (figure 8). This result allows us to use the proposed PCFC method to evaluate the cross-regional CFC between the phase of VIM signal and amplitude of M1 signal while controlling for the phase of M1 signal in the frequency range of 2–31 Hz. To independently validate the directional influence identified by Granger causality, we performed a TE analysis. The results confirmed a significant, unidirectional information flow from M1 to VIM, peaking in the beta frequency range. The information flow in the opposite direction (VIM to M1) was found consistently lower for all frequency bands from 8 to 40 Hz. This finding corroborates the DTF analysis and supports the causal assumption that M1 influences VIM.

**Figure 9.**
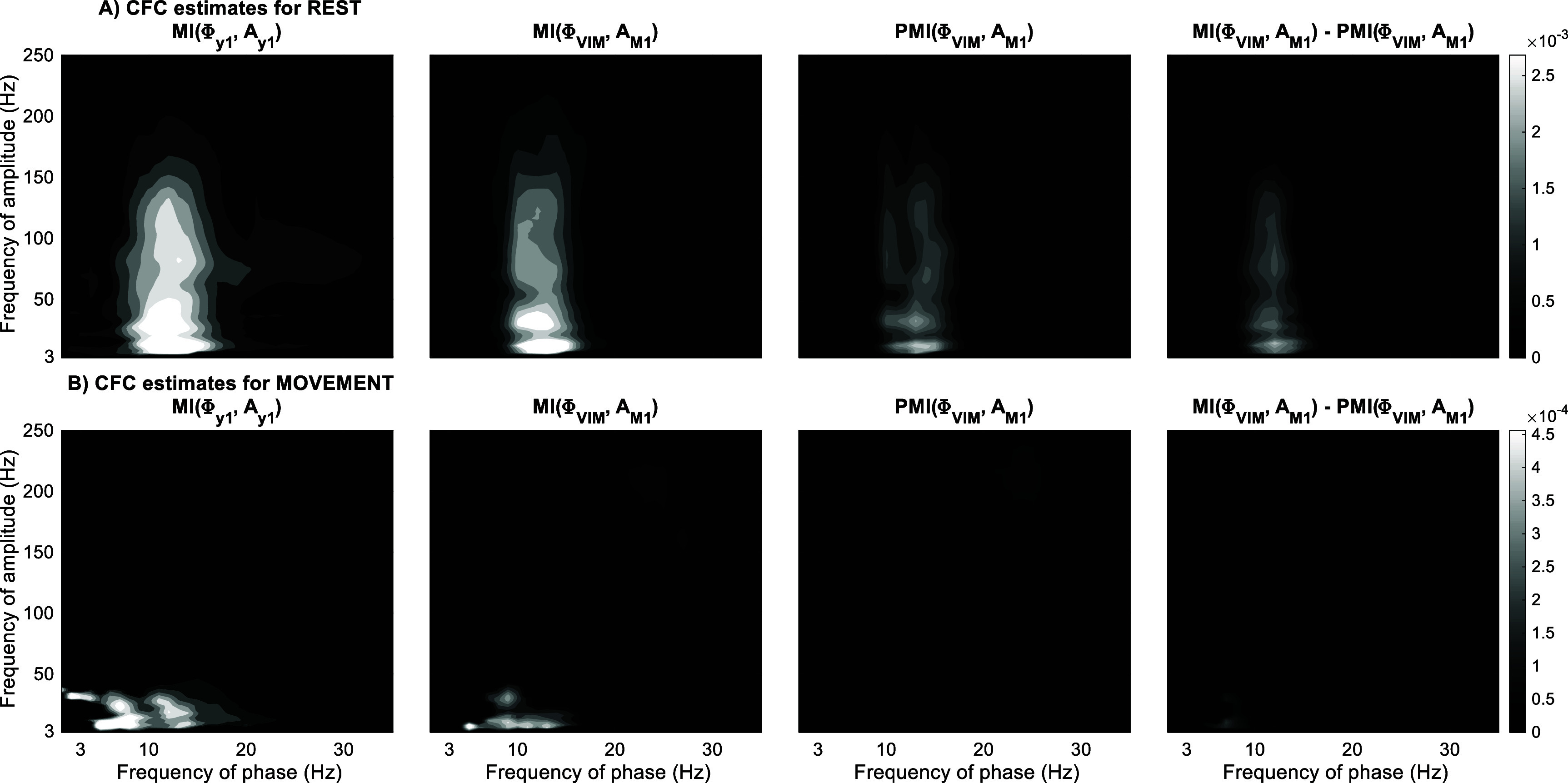
Matrices of CFC estimations for the human dataset based on modulation index and proposed method during rest (A) and movement (B) phases. Insignificant MI values based on nonparametric permutation testing (*p* ⩾ 0.05) and cluster-based correction for multiple comparison have been masked with zero values.

In figure 9(A), significant focal and cross-regional CFC between the phase of 8–18 Hz oscillations (alpha and low beta bands) and amplitude of 8–200 Hz (activity can be observed in M1 and ViM-M1 respectively. ${\text{PMI}}\left( {{\phi _{{\text{VIM}}}},{A_{{\text{M}}1}}} \right)$ is statistically significant in the mentioned frequency ranges however, controlling for ${\phi _{{\text{M}}1}}$ seems to significantly reduce this cross-regional coupling. During the movement condition (figure 9(B)), the significant unbiased cross-regional coupling measured with PMI was attenuated and was no longer statistically significant for the mentioned PAC, consistent with previous reports of the suppression of CFC during movement (Miller *et al* 2012, Yanagisawa *et al* 2012).

## 4. Discussion

CFC continues to be identified as an important measure of brain function, especially as a disease biomarker. For example, a recent report evaluated both within-frequency coupling (WFC) and CFC across brain regions to identify differences between individuals with minimally conscious state vs vegetative state (Yin *et al* 2025). He and colleagues likewise employed cross-network CFC analyses to assess the effect of subconcussive impacts on network dynamics (He *et al* 2025). Similarly, Fang and colleagues reported on the role of thalamofrontal consciousness-related CFC in gating conscious perception (Fang *et al* 2025). While these elegant analysis identify disease-specific changes in oscillatory synchronization across brain regions and in most cases simultaneously assessed cross-site phase synchronization, the reported approaches do not seem to adequately account for the potential for cross-site WFC to result in apparent or artifactual cross-site CFC. The methods reported here seek to disentangle this cascade which may otherwise result in aberrant interpretation of cross-site CFC across brain networks if underlying cross-site phase synchrony is not accounted for.

We propose and evaluate a method to incorporate a confounding bias of a latent node in cross-regional CFC analysis. By incorporating causality analysis, PCFC can be used to draw ‘cause and effect’ conclusions. However, the calculation of PCFC is independent of the causal direction of cross-regional coupling and causal analysis is only used beside PCFC to allow for determination of the confounding bias. For instance, cross-regional CFC between thalamic theta oscillations and cortical beta activity was observed in human LFPs and was shown to be correlated with a causal thalamocortical coherence from cortex to thalamus in theta range. In this case, the nodes of thalamic theta phase, cortical theta phase and cortical beta amplitude formed a chain (as opposed to a fork) in their corresponding DAG. Thus, if we calculate PCFC between thalamic theta phase and cortical beta amplitude while controlling for cortical theta phase, elimination (or reduction) of coupling does not conclude existence of a confounding bias and it could confirm the hypothesis that thalamus regulates cortical activity, at least in part, through thalamocortical PAC.

Given the dynamic nature of CFC it is essential for cross-regional CFC to be sensitive to temporal changes to allow for a second layer of correlation analysis with other measures of coupling. Time sensitive methods such as instantaneous PAC have been used along with other temporal coherence measures to incorporate the dynamicity of CFC, as reported recently by Fang and colleagues (Fang *et al* 2025). However, their temporal resolution may be limited as CFC measures require a certain number of oscillation cycles particularly for LFOs. Neurophysiological recordings especially from subcortical structures of human subjects are only available through invasive clinical interventions such as surgical implantation of deep brain stimulator leads or stereo-electroencephalographic arrays for epilepsy monitoring, which provide a limited window of time for research. This further limits the temporal resolution and statistical power of mentioned multilayer cross-regional CFC. Therefore, we believe PCFC can serve as an integrated method of network level CFC analysis which is sensitive to dynamic variation of couplings. To support this claim, we tested our method using two simulated datasets: one with same-frequency phase coupling and cross-regional and focal PAC happening simultaneously and one with them happening in non-overlapping time segments. Other commonly used methods such as instantaneous PAC can potentially be used along with other time sensitive measures to evaluate the dynamic nature of CFC, however their temporal resolution may be limited as CFC measures require a certain number of oscillation cycles particularly for LFOs.

Beyond theoretical assumptions, a practical concern for such methods is their sensitivity to data quantity, noise, and parameter selection. Our sensitivity analyses directly address these issues. The simulations confirm that PMI is robust against significant noise and performs reliably even with the limited signal durations common in clinical settings. Alternative methods to MI such as the Wasserstein distance-based metric (WMI) have recently been developed which have been shown to be more robust to short data lengths (Ohki 2022). While our proposed method is based on MI, it could be extended to apply to alternative CFC methods using the same do-calculus procedures. Specifically, WMI uses the same preprocessing method as MI and only differs in how the measured amplitude distribution differs from the null hypothesis (i.e. using Wasserstein distance rather than KL divergence). The same approach proposed here could be applied to adjust the amplitude probability to account for a confounder before calculating the Wasserstein distance. Future work can verify the performance of our confounding adjustment procedure in other CFC algorithms.

PCFC was applied to a human dataset between the phase of VIM and amplitude of M1 and it was shown that the M1 phase had a direct causal effect on the VIM phase. Unlike the results for the simulated dataset, PCFC only demonstrated a reduction in coupling rather than an elimination. Even though in our illustration we did not use a DAG with weighted edges to account for the strength of causal couplings, the weight of a causal interaction was incorporated in the conditional probability distributions of the nodes and therefore a reduction (rather than elimination) of PCFC can potentially indicate the partial role M1 phase as the confounding bias in observed VIM-M1 CFC. Controlling for confounders is also limited to the sampling of neural activity recorded by the implanted electrodes which cannot fully represent the heterogeneity of neural activity within and across brain regions. Other unmeasured network nodes can therefore mediate or confound any cross-regional coupling, making it impossible to say for certainty that a direct connection exists. The value of our tool is that it can identify cases where measured nodes explain coupling identified by existing methods, ruling out direct misidentified connections. We should also note that the residual PMI-MI in figure 9 is only to illustrate the significant reduction of PCFC and may not be an appropriate measurement for the partial involvement of the confounding bias especially as MI is a logarithmic measurement by nature.

The current analysis focuses specifically on identifying a confounding node in a relationship between two signals. This relationship was specifically chosen because it is the simplest causal structure that can result in an apparent connection between two regions where no actual causal link exists. Other intermediate nodes such as mediators can have an effect on a causal relationship as well. While they are not directly studied here, the current method can be applied to analyze such relationships as well. For instance, the effect of a d-separating node (i.e. a mediator or confounder) can be identified by controlling for the intermediate node. The current method could then be applied to identify whether the method is a confounder (i.e. check whether ${\text{Pr}}\left( {y|x} \right) = {\text{Pr}}\left( y \right)$). Future work will evaluate this method in identifying other causal relationships.

A potential methodological consideration is the Hilbert transform’s assumption of narrowband signals, a condition often violated by complex physiological data. To address this, we validated our method using simulated LFP-like signals that included realistic broadband and aperiodic noise. The results confirm that PMI remains robust, effectively differentiating true from confounded coupling even with these non-sinusoidal signals, which supports the method’s generalizability to real-world neurophysiological recordings.

While this patient study demonstrates the potential for PCFC to disentangle causal linkages with confounding bias in a clinical setting, it is limited to a single patient and therefore serves as a proof of concept rather than a comprehensive study. Future studies are needed to validate the method across patient populations and in varying clinical contexts.

## 5. Conclusion

Cross-regional CFC analysis addresses the network level interaction among distributed oscillations and focal task-specific activities by probing spatially distinct nodes of the network. PCFC accounts for the potential confounding role of distinct nodes. When used along with same-frequency causal analysis, it can further highlight the casual aspect of network interactions. PCFC can address the need to perform second layers of correlation analysis among different measures of coupling particularly for the temporally limited invasive human data.

## Data Availability

The data that support the findings of this study are openly available at the following URL/DOI: https://dabi.loni.usc.edu/.
